# Genito-Urinary and Syphilitic Diseases

**Published:** 1897-04

**Authors:** Byron H. Daggett

**Affiliations:** Buffalo, N.Y.


### GENITO-URINARY AND SYPHILITIC DISEASES.

CONDUCTED BY BYRON H. DAGGETT, M. D., Buffalo, N. Y.

### TREATMENT OF SYPHILO-DERMATA.

GOTTHEIL, (*Clinical Medicine*, November 25, 1896,) after careful trial of various methods of treating syphilo-dermata, formulates the following conclusions :

1. In the primary stage use calomel locally and no general treatment.

2. As soon as the secondary period is established, internal treatment for mild cases should begin by the administration of $\frac{1}{4}$ to $\frac{3}{4}$ grain of protoiodide of mercury, three times daily, continued for three months or until the symptoms disappear.

In severe cases, characterised by pustular eruptions, anginas, headaches, and the like, a course of six to ten intramuscular injections of five to ten minims of albolin, holding 10 per cent. of calomel (fifty grains to the ounce) in suspension, should be administered at intervals of five to fifteen days.

3. Following this course of injections and cessation of the symptoms of syphilis, tonic treatment should be continued for three months.

4. Then give a second calomel course, as described above, adding fifteen grains of iodide of potassium in milk after meals. This course should be carried out whether there are any secondary manifestations or not.

5. Following this there should be an intermission of specific treatment for six months, unless syphilitic manifestations again appear.

6. In the second year, if marked tertiary lesions appear, give iodide of potassium in increasing doses of sixty to 600 grains daily, as may be necessary, and in addition give the calomel injections ; if the lesions do not reappear, give a mild course of the protoiodide of mercury.

The mercury plaster-mull is the best local treatment of syphilodermata.

---

### ARGONIN IN GONORRHEA.

Dr. J. W. Daniel (*American Therapist*) reports his clinical experiences with this remedy in twenty-six cases. It is claimed that argonin does not irritate the urethral mucous membrane, even in concentrated solution. Argonin is soluble in hot water up to 10 per cent.; it is not decomposed by albuminous substances or precipitated by sodium chloride. Fifteen parts contain as much silver as one part of silver nitrate.

There was no complaint of pain or irritation from any of the twenty-six cases, although twenty of them were in the acute stage. On the contrary, scalding and inflammation rapidly subsided and cordee disappeared within twenty-four hours after its use. In the twenty acute cases the gonococci disappeared entirely in from four to six days. Fifteen grains were used to the ounce of water, and six to eight daily applications were made.

A 5 per cent. solution is fully as efficacious in destroying the gonococci as the stronger solution. The urethral syringe was used for three or four days and then followed by the long-nozzled syringe, and the patient was directed to hold the solution eight or ten minutes.

The discharge ceased about the seventh day in the majority of cases, and the injection was continued for six or eight days as a precautionary measure. As soon as all mucous strings or threads disappeared from the urine, the cure was regarded as complete. There were relapses in two cases, which were readily cured by repetition of the treatment. All uncomplicated cases of acute gonorrhea may be cured in from twelve to eighteen days if this treatment is properly followed out. Dr. Daniel expresses himself as more than satisfied with the results so far obtained from the use of this drug.

---

### EXTERNAL APPLICATION OF PILOCARPINE IN THE TREATMENT OF NEPHRITIS.

Molliere, in 1894, proposed the external use of pilocarpine for the treatment of nephritis. The treatment consists of rubbing into the dorso-lumbar region an ointment composed of three ounces of vaseline, and from three-quarters to one and a half grains of pilocarpine nitrate. This ointment is to be applied every morning and covered with a layer of cotton, which remains on during the day.

Julia (*Lyon Médical*, December 6, 1896,) reports eighty patients treated by this method. Some of these patients had acute nephritis and others chronic nephritis. The chronic cases were much improved ; the acute were rapidly restored to health. During the treatment the albumin often entirely disappeared. These inunctions produced marked diaphoresis and diuresis. Brouowski alleges that strontium lactate, given in six daily doses of fifteen grains each, is the most valuable remedy in the treatment of acute inflammatory conditions of the kidney. It appears that, like many other conditions, there is more than one way of successfully treating acute nephritis.

